# Thyroid hormone levels are associated with metabolic components: a cross-sectional study

**DOI:** 10.3325/cmj.2020.61.230

**Published:** 2020-06

**Authors:** Ante Punda, Veselin Škrabić, Vesela Torlak, Ivana Gunjača, Vesna Boraska, Perica, Ivana Kolčić, Ozren Polašek, Caroline Hayward, Tatijana Zemunik, Antonela Matana

**Affiliations:** 1 Clinical Department of Nuclear Medicine, University Hospital Split, Split, Croatia; 2 Clinical Department of Pediatrics, University Hospital Split, Split, Croatia; 3 Department of Medical Biology, University of Split, School of Medicine, Split, Croatia; 4 Department of Public Health, University of Split School of Medicine, Split, Croatia; 5 MRC Human Genetics Unit, Edinburgh, United Kingdom; 6 Department of Mathematics, Faculty of Science Split, Split, Croatia

## Abstract

**Aim:**

To analyze the association of thyroid function and hormone levels with metabolic syndrome (MetS) and its components.

**Methods:**

This cross-sectional population-based study involved 2183 Croatian individuals with no history of thyroid disease, hypertension, diabetes, and hyperlipidemia. MetS was diagnosed according to the National Cholesterol Education Program’s Adult Treatment Panel III criteria.

**Results:**

We found no association between thyroid function groups and the prevalence of MetS and its components. Clinically hypothyroid participants showed significantly higher triceps skinfold measurements than subclinically hypothyroid and euthyroid participants. Furthermore, clinically hypothyroid participants had higher abdominal skinfold thickness than subclinically hypothyroid participants. Otherwise, suprailiac and abdominal skinfold measurements were higher in the subclinically and clinically hyperthyroid group of participants compared with euthyroid and subclinically hypothyroid participants. A strong positive association of thyroid-stimulating hormone (TSH) and strong negative association of free triiodothyronine (fT3) and free thyroxine (fT4) levels with HOMA-IR and cholesterol levels were found. Furthermore, the fT4 level also showed a strong negative association with HDL and triceps skinfold thickness.

**Conclusions:**

This study supports the standing that TSH, fT3, and fT4 levels are important variables to determine the association of thyroid function with MetS.

Metabolic syndrome (MetS) is a medical condition that results from over-nutrition and a sedentary lifestyle (1). It is one of the most frequent endocrine disorders characterized by a cluster of metabolic abnormalities including obesity, dyslipidemia, hyperglycemia, and hypertension (2). The presence of MetS has been closely linked to an increased risk of developing cardiovascular diseases and type-2 diabetes (1,3). MetS has been differently defined by different associations: the National Cholesterol Education Program’s Adult Treatment Panel (NCEP-ATP) III, the International Diabetes Federation, the Chinese Diabetes Society, and the Joint Interim Statement (4). These definitions slightly differ in the components used to define MetS, which mainly results from the variation in the prevalence of MetS in different populations (4). The most commonly used criteria, NCEP-ATP III, include elevated fasting plasma glucose, increased waist circumference, hypertriglyceridemia, low serum high-density lipoprotein (HDL) cholesterol, and hypertension. At least three of these components have to be present for the diagnosis of MetS (5,6).

Serum levels of thyroid hormones (THs) have been associated with MetS components since they target the same metabolic pathways (5,7). THs are of major importance for metabolism and energy balance. Increased and decreased THs concentrations lead to insulin resistance, influence glucose and lipid metabolism, and consequently induce or aggravate some parameters of MetS (8). Hypothyroidism and hyperthyroidism are linked to atherosclerotic cardiovascular disease, which is explained by the influence of THs on lipid metabolism and increased blood pressure (5).

In recent years, several studies have analyzed the relationship between thyroid dysfunction and MetS components (1,6,9,10). The reported results are conflicting, and many discrepancies have been observed. Therefore, the aim of this study was to assess the association between thyroid function and hormone levels with MetS and its components in our population of individuals without a history of thyroid disorders, hypertension, diabetes, and hyperlipidemia. We also assessed the characteristics of the study population relevant to MetS by thyroid function group and analyzed the association between THs levels and metabolic parameters.

## Methods

### Study population

This cross-sectional study was performed on samples from three Croatian populations: the mainland city of Split and the islands of Vis and Korčula. The samples were obtained from the large-scale “10,001 Dalmatians” biobank project (11). The participants were adult volunteers aged 18-93 years from the general population. We excluded participants with known thyroid pathologies who had undergone thyroid surgery or were taking thyroid medication, as well as those who received dyslipidemia medications, blood pressure regulators, insulin, or glucose regulators and those who reported hypertension, hyperlipidemia, and diabetes. The final sample comprised of 2183 eligible participants. The written informed consent was obtained from all participants, and the Ethics Committee of the University of Split School of Medicine approved the study protocol (2181-198-03-04-14-0031).

### Laboratory measurements

Circulating thyroid hormone and antibody levels in the plasma were determined by immunoassay methods in the Laboratory of Biochemistry in the Department of Nuclear Medicine at the University Hospital Split. Plasma concentrations of TSH, free triiodothyronine (fT3), free thyroxine (fT4), thyroglobulin autoantibodies (TgAb), and thyroid peroxidase antibodies (TPOAb) were measured with a “Liason” Biomedica Chemiluminescence Analyzer (DiaSorin, Saluggia, Italy) using *in vitro* assays for the quantitative determination of thyroid hormone and antibody levels. Plasma samples were collected during recruitment and stored at -80°C, while thyroid hormones and antibodies were determined subsequently. The reference ranges for our population are 0.3-3.6 mlU/L for TSH, 3.39-6.47 pmol/L for fT3, 10.29-21.88 pmol/L for fT4, 5-100 IU/mL for TgAb, and 1-16 IU/mL for TPOAb levels.

The biochemical analyses of HDL, low-density lipoproteins, total cholesterol, triacylglycerols (TG), and blood glucose were performed according to standard internationally accepted procedures and are described in detail elsewhere (12).

The insulin concentration was determined with the Roche E170 electrochemiluminescence immunoassay (ECLIA) (Basel, Switzerland) following standard procedures (13). Biochemical measurements were performed on serum samples collected at the time of recruitment.

### Assessment of other variables

The mean value of the two measurements of both systolic and diastolic blood pressure was used. The waist circumference was measured using standard methods (14). Triceps, subscapular, suprailiac, and abdominal skinfold thickness were measured using calipers. The mean value of three measurements of each skinfold thickness was used in the analyses.

### Definitions

Euthyroidism was defined as TSH (reference range 0.3-3.6 mlU/L) and fT4 (reference range 10.29-22.7 pmol/L) within the reference range. Subclinical hypothyroidism was defined as TSH>3.6 mlU/L and fT4 within the reference range, while clinical hypothyroidism was defined as TSH>3.6 mlU/L and fT4 < 10.29 pmol/L. Subclinical hyperthyroidism was defined as TSH<0.3 mlU/L and fT4 within the reference range, while clinical hyperthyroidism was defined as TSH<0.3 mlU/L and fT4 > 22.7 pmol/L. Metabolic syndrome was defined as the presence of three or more of the following criteria: 1) waist circumference >102 cm in men and >88 cm in women; 2) HbA1C≥5.7 (%); 3) systolic blood pressure (SBP)>130 mm Hg or diastolic blood pressure (DBP)>85 mm Hg; 4) TG≥1.7 mmol/L; 5) HDL≤1.04 mmol/L in men and ≤1.29 mmol/L in women.

The homeostasis model assessment index for insulin resistance (HOMA-IR) was calculated as fasting insulin (mU/L) times fasting glucose (mmol/L) divided by 22.5 (15). Body mass index (BMI) was calculated by dividing the weight in kilograms by the height in meters squared.

### Statistical analysis

The Kolmogorov-Smirnov test was used for normality checking. Non-normally distributed continuous variables are expressed as medians with lower and upper quartiles, and categorical variables as frequencies (percentages) (Tables 1 and 2). The Kruskal-Wallis test with *post hoc* analysis according to Dunn or the χ^2^ test for dichotomous variables were used to assess if there are significant differences between the groups. To assess the differences in the prevalence of MetS and its components among different groups of thyroid function, we used univariate logistic regression analysis with the thyroid function group as an independent variable. We also performed multivariate linear regression analyses with TSH, fT3, and fT4 levels as dependent variables, and BMI, waist circumference, total cholesterol, triacylglycerols, HDL, systolic and diastolic blood pressure, HOMA-IR, and skinfold thickness measurements as independent variables. The level of significance was set at *P* < 0.05. Statistical analyses were performed with SAS software (SAS Institute, Cary, NC, USA).

**Table 1:** Characteristics of the study population by thyroid function group*

	Subclinically hypothyroid participants (N = 159)	Clinically hypothyroid participants (N = 49)	Euthyroid participants (N = 1959)	Subclinically and clinically hyperthyroid participants (N = 16)	p^†^
Age	50 (34, 61)	60 (52, 67)	52 (42, 63)	50 (36, 70)	0.0004
Sex					
M	59 (37.11)	6 (12.24)	805 (41.09)	7 (43.75)	0.0006
F	100 (62.89)	43 (87.76)	1154 (58.91)	9 (56.25)	
Body mass index (kg /m^2^)	26.22(23.58,29.25)	27.55(25.46,29.81)	26.76(23.96,29.84)	27.7(24.54, 29.17)	0.16
Waist circumference (mm)	901.5 (830,990)	941 (850,990)	932 (840,1010)	950 (805,994)	0.11
Triceps skinfold (mm)	18.60 (12.9,23.7)	24.7 (19.6,29.8)	19.1(12.9, 24.9)	23.35(16.3,30.15)	<0.0001
Subscapular skinfold (mm)	16.7(12.3, 23.3)	19.9 (15.6, 26.3)	18.25 (13.9, 24.4)	23.3 (14.7, 32.35)	0.0117
Suprailiac skinfold (mm)	21.9 (14.1, 32.5)	25.1 (18.2, 36)	24.1(16.7, 34.8)	40.85(36.45, 44.85)	0.0001
Abdominal skinfold (mm)	28.6 (20.7, 39.7)	34.7 (27, 44)	32 (22, 43.2)	42.5 (36.95, 48.7)	0.0023
Total cholesterol (mmol/L)	5.4 (4.9, 6.5)	5.7 (5, 6.8)	5.5 (4.8, 6.4)	4.6 (3.7, 6.4)	0.0498
High-density lipoprotein (mmol/L)	1.29 (1.09, 1.54)	1.31 (1.105, 1.53)	1.25 (1.1, 1.5)	1.23 (1.13, 1.35)	0.74
Low-density lipoprotein (mmol/L)	3.6 (2.9, 4.3)	3.6 (2.9, 4.7)	3.6 (2.9, 4.3)	2.7 (2.1, 4.3)	0.08
Triacylglycerols (mmol/L)	1.14 (0.89, 1.7)	1.26 (1, 1.8)	1.24 (0.9, 1.8)	1.3 (1, 1.43)	0.48
Glucose (mmol/L)	5.2 (4.8, 5.8)	5.4 (4.9, 5.8)	5.3 (4.8, 5.7)	4.9 (4.6, 5.5)	0.49
Hemoglobin A1c (%)	5.2 (5, 5.5)	5.4 (5.2, 5.7)	5.3 (5, 5.6)	5.05 (4.9, 5.5)	0.07
Insulin	5.68 (4.15, 8.19)	6.51 (4.38, 10.28)	5.48 (4, 8)	7 (5, 10)	0.23
Homeostasis model assessment index for insulin resistance	1.29 (1.03, 2.004)	1.47 (0.98, 2.71)	1.31 (0.88, 2.06)	1.58 (1.16, 2.04)	0.41
Systolic blood pressure (mmHg)	130 (117.5, 144)	132.5 (122.5, 145)	129 (117, 145)	127.5 (115, 155)	0.80
Diastolic blood pressure (mmHg)	76 (69.5, 84)	80 (71.5, 85)	79 (72, 85)	77.5 (62.5, 85)	0.23
Free triiodothyronine (fT3) (pmol/L)	4.4 (4.1, 4.9)	3.5 (3.1, 3.9)	4.5 (4.3, 4.9)	5.7 (4.9, 6.7)	<0.0001
Thyroglobulin autoantibodies (TgAb) (IU/mL)	13.8 (5, 87.3)	27.15 (7.65, 331)	9.4 (5.5, 19.1)	18.95 (6.4, 323.5)	<0.0001
Positive TgAb	36 (22.6)	19 (38.78)	227 (11.59)	5 (31.25)	<0.0001
Negative TgAb	123 (77.36)	30 (61.22)	1732 (88.41)	11 (68.75)	<0.0001
Thyroid peroxidase antibodies (TPOAb) (IU/mL)	8.7 (2.4, 90.10)	20.5 (4.2, 311)	4.4 (1.7, 10.7)	9.9 (2, 290.75)	<0.0001
Positive TPOAb	58 (36.48)	27 (55.1)	368 (18.79)	7 (43.75)	<0.0001
Negative TPOAb	101 (63.52)	22 (44.9)	1591 (81.21)	9 (56.25)	<0.0001

*Quantitative variables are expressed as median, lower and upper quartiles, while categorical variables are expressed as frequencies and percentages.

†Kruskal-Wallis test or χ^2^ test for dichotomous variables.

**Table 2:** Sex differences in waist circumference and skinfold thickness measurements among patients with different thyroid function*

	Euthyroid patients	Subclinically hypothyroid patients	Clinically hypothyroid patients	Subclinically and clinically hyperthyroid participants	p†
**Women**					
Waist circumference	861 (795, 933)	934 (847, 985)	890 (804, 980)	854 (790, 994)	0.07
Triceps skinfold	21.85 (18.2, 26.3)	26 (20,30.7)	22.7 (18.2, 27.5)	26.2 (23.3, 32.1)	0.04
Subscapular skinfold	16.95 (12.1, 24.35)	20.3 (15.8, 29.4)	18.7 (13.7, 25.1)	20 (14.1, 30.5)	0.01
Suprailiac skinfold	21.3 (13.8, 32.35)	25.2 (18.3, 38.3)	24 (16.6, 34.8)	39.2 (32.8, 43.9)	0.01
Abdominal skinfold	28.1(20.9, 38.75)	35.8 (29.1, 44.2)	31.9 (21.6, 43.3)	40.5 (38.1, 46.6)	0.01
**Men**					
Waist circumference	960 (909.5, 1019.5)	980.5 (950, 1040)	972 (915, 1050)	955.5 (950, 962)	0.57
Triceps skinfold	12.8 (9.3, 16.1)	17.25 (11.2, 21.6)	12.5 (9.6, 17.6)	17.7 (14.9, 23.4)	0.06
Subscapluar skinfold	16.5 (13.1, 20)	15.1 (14.2, 20.3)	17.9 (14, 23.2)	27.1 (21.3, 38.4)	0.04
Suprailiac skinfold	22.5 (15.7, 34.2)	21.15 (16.7, 34.7)	24.2 (16.8, 34.8)	42.5 (39, 59.1)	0.007
Abdominal skinfold	29.1 (20.7, 40.6)	29.25 (25.5, 34.4)	32.1 (22.6, 42.6)	47 (35.8, 80)	0.09

*All variables are expressed in millimeters.

**†**Kruskal-Wallis test.

## Results

The study involved 2183 individuals, 1306 (59.83%) of them women and 877 (40.17%) men, with the median age of 52 and 54, respectively (IQR 20, 24, respectively). Among the participants, 1959 (89.74%) were euthyroid, 159 (7.3%) subclinically hypothyroid, 49 (2.24%) clinically hypothyroid, while 16 (0.73%) were clinically and subclinically hyperthyroid (2 clinically and 14 subclinically hyperthyroid) (Table 1). MetS was present in 569 (26.07%) individuals. Clinically hypothyroid participants were older than subclinically hypothyroid (*post hoc* *P* = 0.0004). Women had a higher prevalence of clinical and subclinical hypothyroidism.

Clinically hypothyroid participants had higher triceps skinfold thickness than subclinically (*P* = 0.0001) and euthyroid participants (*P* = 0.0002). Furthermore, clinically hypothyroid participants had higher abdominal skinfold thickness than subclinically hypothyroid participants (*P* = 0.032). The clinically and subclinically hyperthyroid group had higher suprailiac and abdominal skinfold thickness than subclinically hypothyroid and euthyroid participants (suprailiac: *P* = 0.0002, *P* = 0.0009, respectively; abdominal *P* = 0.0081, *P* = 0.0394, respectively). Clinically hypothyroid participants had higher TgAb and TPOAb, and lower fT3 values than euthyroid participants (*post hoc* *P* = 1.9^e-5^, *P* = 3.6^e-8^, *P* = 2^e-16^ for TgAb, TPOAb, and fT3, respectively) and lower fT3 values than subclinically hypothyroid participants (*P* < 0.0001). In addition, clinically hypothyroid participants had a higher prevalence of positive TgAb and TPOAb than subclinically hypothyroid and euthyroid participants (Table 1).

Additionally, we tested the sex differences in waist circumference and skinfold thickness among groups with different thyroid function (Table 2). Clinically hypothyroid women had higher waist circumferences, and triceps and abdominal skinfold thickness than subclinically hypothyroid women (*post hoc* *P* = 0.04, *P* = 0.04, *P* = 0.01, respectively). Subclinically hypothyroid men had lower suprailiac skinfold thickness than clinically and subclinically hyperthyroid men (*P* = 0.006) (Table 2).

No significant association was observed between thyroid function groups and the prevalence of MetS (*P* = 0.7) (32.65% clinically hypothyroid, 26.29% euthyroid, and 22.64% subclinically hypothyroid participants had MetS). No significant association was detected between the prevalence of other MetS components and thyroid function groups (Table 3).

**Table 3:** The differences in the prevalence of metabolic syndrome and its components among different groups of thyroid function as assessed by univariate logistic regression analysis

	Parameter estimate	Odds ratio (95% confidence interval)	*P*
Metabolic syndrome			
subclinically hypothyroid	-0.0754	0.651 (0.198, 1.858)	0.7
clinically hypothyroid	-0.1056	0.631 (0.18, 1.971)	0.7
euthyroid	-0.1733	0.590 (0.185, 1.614)	0.3
subclinically and clinically hyperthyroid	-	-	-
Abdominal obesity			
subclinically hypothyroid	0.0324	0.825 (0.287, 2.224)	0.9
clinically hypothyroid	-0.1439	0.692 (0.224, 2.022)	0.5
euthyroid	-0.1128	0.714 (0.257, 1.842)	0.4
subclinically and clinically hyperthyroid	-	-	-
Hypertriglyceridemia			
subclinically hypothyroid	-0.1176	0.672 (0.205, 1.908)	0.5
clinically hypothyroid	-0.0487	0.72 (0.205, 2.250)	0.8
euthyroid	-0.1141	0.674 (0.213, 1.834)	0.5
subclinically and clinically hyperthyroid	-	-	-
Reduced high-density lipoprotein			
subclinically hypothyroid	0.0144	1.074 (0.403, 2.783)	0.9
clinically hypothyroid	0.1159	1.188 (0.409, 3.384)	0.6
euthyroid	-0.0738	0.983 (0.384, 2.441)	0.6
subclinically and clinically hyperthyroid	-	-	-
Hyperglycemia			
subclinically hypothyroid	-0.0854	0.761 (0.232, 2.16)	0.7
clinically hypothyroid	0.0564	0.877 (0.247, 2.773)	0.8
euthyroid	-0.1582	0.708 (0.223, 1.919)	0.3
subclinically and clinically hyperthyroid	-	-	-
Hypertension			
subclinically hypothyroid	-0.1169	0.748 (0.271, 1.98)	0.5
clinically hypothyroid	-0.00609	0.835 (0.279, 2.415)	0.9
euthyroid	-0.0507	0.799 (0.3, 2.032)	0.7
subclinically and clinically hyperthyroid	-	-	-

Since TSH, ft3, and fT4 were not normally distributed, the variables were logarithmically transformed to obtain normally distributed variables. Multiple linear regression analysis was performed with logTSH, logfT3, and logfT4 variables as response variables and metabolic parameters listed in Table 3 as predictor variables. After observing the full regression model for logTSH, as well as the models produced by forward, backward, and stepwise selection, we concluded that our full model (adjusted R^2^ = 0.0265, *P* < 0.0001) explains the variability of the data just as good as the models obtained by forward (adjusted R^2^ = 0.0295, *P* < 0.0001), backward (adjusted R^2^ = 0.0278, *P* < 0.0001), and stepwise (adjusted R^2^ = 0.0287, *P* < 0.0001) selection processes.

Multiple linear regression model was also significant for logft3 (adjusted R^2^ = 0.0169, *P* = 0.0006) and for logfT4 (adjusted R^2^ = 0.0353, *P* < 0.0001).

The results showed that logTSH, and therefore TSH, were positively associated with HOMA-IR (*P* < 0.0001) and cholesterol level (*P* = 0.0325). On the other hand, logft3, and therefore ft3, and logft4, and therefore ft4, were negatively associated with HOMA-IR (*P* = 0.0018 and *P* = 0.0023, respectively) and cholesterol (*P* = 0.0007 and *P* = 0.0038, respectively). Ft4 was also negatively associated with HDL (*P* = 0.0085) and triceps skinfold thickness (*P* = 0.0378) (Table 4).

**Table 4:** Association of thyroid stimulating hormone (TSH), free triiodothyronine (fT3), and free thyroxine (fT4) levels with metabolic characteristics*

	TSH		fT3		fT4	
Variable	β (95% CI)	p	β (95% CI)	p	β (95% CI)	p
Body mass index	0.0024 (-0.015, 0.0198)	0.787	-0.00003 (-0.0038, 0.0037)	0.987	-0.0043 (-0.0091, 0.0004)	0.074
Waist circumference	0.0001 (-0.0007, 0.0004)	0.701	0.00006 (-0.00006, 0.0002)	0.327	0.0001 (-0.00003, 0.0003)	0.119
Total cholesterol	0.0400 (0.0033, 0.0767)	0.032	-0.0137 (-0.0217, -0.0058)	0.0007	-0.0148 (-0.0248, -0.0047)	0.003
Triacylglycerols	-0.0312 (-0.0842, 0.0216)	0.246	0.0032 (-0.0082, 0.0147)	0.578	-0.00205 (-0.01652, 0.01243)	0.781
High-density lipoprotein	-0.1317 (-0.2715, 0.0079)	0.064	0.0097 (-0.0204-0.0399)	0.525	-0.0513 (-0.0896, -0.0131	0.008
Systolic blood pressure	0.0019 (-0.0005, 0.0043)	0.121	-0.00009 (-0.0006, 0.0004)	0.730	5.67^e-7^ (-0.0006, 0.0006)	0.998
Diastolic blood pressure	-0.0028 (-0.0079, 0.0023)	0.279	-0.0002 (-0.0014, 0.0008)	0.615	-0.0001 (-0.0015, 0.0012)	0.850
Homeostasis model assessment index for insulin resistance	0.0138 (0.0092, 0.0185)	<0.0001	-0.0016 (-0.0026, 0.0005)	0.001	-0.0019 (-0.0032, -0.0007)	0.002
Triceps skinfold	0.0013 (-0.0034, 0.0062)	0.577	-0.0008 (-0.0018, 0.0002)	0.120	-0.0014 (-0.0027, -0.00007)	0.037
Subscapular skinfold	-0.0021 (-0.0078, 0.0036)	0.476	-0.0005 (-0.0017, 0.0007)	0.419	-0.0004 (-0.0026, 0.0011)	0.550
Suprailiac skinfold	0.0011 (-0.0031, 0.0054)	0.597	-0.0005 (-0.0014, 0.0004)	0.267	0.0004 (-0.0007, 0.0016)	0.458
Abdominal skinfold	0.0011 (-0.0023, 0.0046)	0.527	0.0006 (-0.0001, 0.0013)	0.104	0.0004 (-0.0005, 0.0013)	0.406

*β – effect size; CI – confidence interval.

## Discussion

The main finding of this study is a strong positive association of TSH and a strong negative association of fT3 and fT4 levels with HOMA-IR and cholesterol levels in individuals without a history of thyroid disorders. Furthermore, the fT4 level also showed a strong negative association with HDL and triceps skinfold. No association was observed between thyroid function groups and the prevalence of MetS. Differences in skinfold thickness were observed between groups and they showed the same directions in women after the sex-stratified analysis was performed.

The observed prevalence of subclinical hypothyroidism of 7.3% in our study is in accordance with the previously reported prevalence of 4%-10% in the adult population (16). The prevalence of clinical hypothyroidism was 2.24%, which is in agreement with the published findings for the general population of up to 4.2% for men and up to 13.3% for women (17). The prevalence of MetS in the United States increased over time from approximately 30.07% (1988-1994) to 35% (1999-2002) (2). In this study, we observed a prevalence of 26.07%.

The HOMA-IR is a mathematical method that measures insulin resistance using fasting serum insulin and fasting plasma glucose values (18). It is an important predictor of MetS, and its measurement is mandatory since insulin resistance has become a key component of MetS (19). Several cross-sectional studies have reported a positive association between TSH levels and MetS in different populations (3,19-22). Only a few previous studies analyzed the association between insulin resistance and TSH levels (20,23-25). In accordance with our findings, Garduno-Garcia et al (24), Bensenor et al (20), and Mahran et al (25) reported a positive correlation between TSH levels and HOMA-IR. The positive association of TSH and cholesterol levels observed in this study is also in accordance with the findings of Garduno-Garcia et al (24). On the other hand, our results are not consistent with those of Lai et al (23), who observed no association between TSH levels within the reference range and HOMA-IR in an adult Chinese population. Several studies described an association between low fT4 levels and increased insulin resistance (1,24,25). Furthermore, the negative association of fT4 with HOMA-IR found in this study was also observed in a Hispanic population (24). These findings are expected due to a negative feedback mechanism in endocrine regulation when decreased fT4 is followed by higher TSH levels (3). Both hypo- and hyperthyroidism are often accompanied by disturbed glucose transport and the development of insulin resistance (26,27). Hyperthyroidism involves the decreased expression of glucose transporters (GLUT) in peripheral tissues (27). Although impaired translocation of GLUT4 on the monocyte plasma membrane was observed in patients with hypothyroidism, it seems that some additional mechanisms are involved in the development of insulin resistance (26). One of these mechanisms could be poor blood circulation in peripheral tissue (26). Furthermore, insulin and T3 have a synergistic effect on glycemic control since they have similar action sites in achieving glucose homeostasis on a cellular and molecular level (26,28). This synergism seems to regulate glycolysis, gluconeogenesis, and glycogenolysis (28).

We also found a negative association between the triceps skinfold thickness and fT4 levels. Triceps skinfold measurements were higher in clinically hypothyroid participants compared with euthyroid and subclinically hypothyroid participants. Even more, clinically hypothyroid participants had higher abdominal skinfold measurements compared with subclinically hypothyroid participants. It is known that thyroid status is related to weight change and that serum TSH positively correlates with BMI (29). A review article by Pearce et al (29) showed 0.9 kg increase in weight and a 0.3 kg/m^2^ increase in BMI in women for every 1 mIU/L increase in baseline TSH serum, and 0.8 kg increase in weight and 0.2 kg/m2 in BMI in men. The same article states that thyroid status may influence adipose tissue distribution and that fT4 is independently and inversely associated with visceral fat stores (29). Furthermore, the main center for the control of food intake is located in the hypothalamus, which interacts with the thyroid axis control (8). Remarkably, in our study, suprailiac and abdominal skinfold measurements were higher in the subclinically and clinically hyperthyroid participants compared with subclinically hypothyroid and euthyroid participants. The discrepancy observed in the subclinically and clinically hyperthyroid group in this study can be explained by the presence of participants who were observed as subclinically hyperthyroid with still undefined phenotype, since conversion from hyperthyroidism to hypothyroidism is not a rare phenomenon. Sometimes, hypothyroidism or Hashimoto's disease can occur following the Graves' disease episode due to extended immune response in Graves' disease (30). It is probably induced with the immune response to thyroid antigens, ie, TPOAb and TgAb, which may stimulate lymphocyte infiltration and finally develop Hashimoto's thyroiditis (30).

The sex-stratified analysis showed that the differences in skinfold measurements had the same direction in women. Clinically hypothyroid women showed higher waist circumferences and triceps and abdominal skinfold thickness than subclinically hypothyroid women. Some studies observed a positive correlation between TSH levels and leptin, possibly because leptin, a protein produced by adipocytes, can stimulate thyrotropin-releasing hormone (TRH) expression (8,31). Previous studies demonstrated that abdominal obesity and increased waist circumference were less prevalent among men (1,32,33). Leptin is almost exclusively secreted by adipose tissue and can stimulate thyroid axis regulation, consequently stimulating TSH secretion. We can thus conclude that a large number of women in the clinical hypothyroid group contributed to the sex-stratified results of this study.

This study found no association between thyroid function groups and the prevalence of MetS. We also observed no association between the groups in MetS components. A study from central Mexico showed that the euthyroid and subclinical hypothyroid group had an equal prevalence of MetS, which aligns with our findings (24). Contrary to our findings, a small Bangladeshi study (34) showed a higher prevalence of MetS in the hypothyroid compared with the euthyroid group. Recently, a large population study performed on 5422 participants from Tehran showed that clinically hypothyroid individuals had the highest prevalence of MetS, compared with subclinically hypothyroid, euthyroid, and subclinically and clinically hyperthyroid individuals (10).

The strength of the current study is a relatively large sample size and the participants originating from an iodine sufficient area. The limitation of our study is its cross-sectional design, which restricts the possibility to determine a cause-effect relationship between the observed associations. Furthermore, diet and exercise during the day could have influenced the examined variables. We also must emphasize that the study was performed in a middle-aged population (mean age 52, 50, 60, and 50 years in euthyroid, subclinical, clinical hypothyroid, and subclinically and clinically hyperthyroid groups, respectively).

In conclusion, this study extends the existing knowledge that THs levels are important variables related to some metabolic parameters of MetS in individuals without a history of thyroid disorders. We found a positive association between TSH levels and HOMA-IR and the cholesterol levels, while fT3 and fT4 showed a negative association with these parameters. Despite this, we did not find an association between thyroid function groups and the prevalence of MetS. THs levels seem to be convenient parameters to establish the association of thyroid function with MetS.
